# Interaction Between Liver Metabolism and Gut Short-Chain Fatty Acids via Liver–Gut Axis Affects Body Weight in Lambs

**DOI:** 10.3390/ijms252413386

**Published:** 2024-12-13

**Authors:** Haibo Wang, Jinshun Zhan, Shengguo Zhao, Haoyun Jiang, Haobin Jia, Yue Pan, Xiaojun Zhong, Junhong Huo

**Affiliations:** 1 Institute of Animal Husbandry and Veterinary, Jiangxi Academy of Agricultural Science, Nanchang 330200, China; wanghaibo8815@163.com (H.W.); zhanjinshun1985@163.com (J.Z.); jianghaoyun1995@163.com (H.J.); jiahaobin@jxaas.cn (H.J.); py13782525871@163.com (Y.P.); zhongcaoyangchu@163.com (X.Z.); 2 Jiangxi Province Key Laboratory of Animal Green and Healthy Breeding, Institute of Animal Husbandry and Veterinary, Jiangxi Academy of Agricultural Science, Nanchang 330200, China; 3 College of Animal Science and Technology, Gansu Agricultural University, Lanzhou 730070, China; zhaosg@gsau.edu.cn

**Keywords:** Hu lamb, metabolome, VFAs, microbiota, liver–gut axis

## Abstract

The gut–liver axis and its interactions are essential for host physiology. Thus, we examined the jejunal microbiota, fermentation parameters, digestive enzymes, morphology, and liver metabolic profiles in different growth development lambs to investigate the liver–gut axis’s role in their development. One hundred male Hu lambs of similar birth weight and age were raised under the same conditions until they reached 180 days of age. Subsequently, the eight lambs with the highest (HADG) and lowest (LADG) average daily weight gains were slaughtered for index assessment. The study indicates that the body weight, carcass weight, propanoic acid, butyric acid, propanoic acid ratio, butyric acid ratio, and digestive enzymes (beta-glucosidase, microcrystalline cellulase, xylanase, and carboxymethyl cellulase) were significantly higher in HDAG lambs than in LADG lambs (*p* < 0.05). Additionally, there were no significant differences in the jejunal microbiota’s structure and function among lambs at different growth development stages (*p* > 0.05). Overall, our analysis revealed that HADG lambs compared to LADG lambs exhibited an up-regulation of metabolites (such as spermine, cholic acid, succinic acid, betaine, etc.) that were positively correlated with the butyric acid ratio, propanoic acid ratio, propanoic acid, xylanase, microcrystalline cellulase, beta-glucosidase, amylase, carboxymethyl cellulase, carcass weight, and body weight, while these metabolites were negatively correlated with the kidney, acetic acid, acetic acid/ propanoic acid, and acetic acid ratio. Furthermore, there was a significant correlation between liver metabolism and jejunal microbiota. This study revealed significant differences in hepatic metabolites and jejunal fermentation among lambs at different growth stages, which may inform targeted regulation strategies to enhance lamb productivity.

## 1. Introduction

A sheep (*Ovis aries*) is a key livestock species that has developed breeds through natural and artificial selection since the Neolithic era [1]. Sheep rely on gastrointestinal microbiota to convert plant feeds like straw, hay, silage, and grass into meat, wool, fur, and milk [2,3,4]. Meanwhile, gut microbiota, known as the “second genome”, plays an important role in environmental adaptation, homeostasis, and regulating animal performance, including productivity and meat quality, as well as host immunity and digestive metabolism [4,5,6]. Furthermore, the optimal growth and development of young ruminants significantly influence the establishment of their physiological functions, as well as their productive and reproductive performances and overall health status in adulthood [7,8]. Research has demonstrated a significant correlation between rumen microbiota, rumen fermentation, and growth performance in young goats [9]. Additionally, nutritional homeostasis is regulated by the microbiota and the host’s metabolic system, with the liver central to metabolizing carbohydrates, lipids, proteins, and amino acids [10,11]. Therefore, we hypothesize that gut volatile fatty acids (VFAs), microbiota, and metabolites interact within the enterohepatic cycle and may influence animal growth and development [9,12,13,14].

Furthermore, metabolites from specific gastrointestinal microbiota, such as VFAs, bile acids, etc., link microbiota and host interactions, affecting metabolism, immune responses, and gut microbial structure [14,15,16,17]. Research has demonstrated that bile acids are composed of primary and secondary bile acids, among which primary bile acids are synthesized from cholesterol in the liver via either classically or alternatively mediated pathways [18], acting as signaling molecules that impact glucose homeostasis, lipid metabolism, and energy expenditure [17,19]. Additionally, the fermentation of gut microbiota produces VFAs, which are vital for regulating bile acid distribution, preserving the integrity of the gut barrier, influencing liver and pancreatic metabolisms, and supporting host health [16,17]. Research indicates that VFAs can raise the adenosine monophosphate (AMP) concentration and adenosine monophosphate/adenosine triphosphate (AMP/ATP) ratio in skeletal muscle, activate AMP-activated protein kinase (AMPK), promote proliferator-activated receptor gamma coactivator 1α (PGC1α) phosphorylation, enhance fatty acid uptake and oxidation, increase glucose uptake and production, and inhibit lipogenesis and glycolysis [20]. Interestingly, increasing the serum VFA concentration can improve glucose tolerance in patients with type 2 diabetes [21], and butyrate has a significant effect on the blood glucose level of sheep, while this effect is strongly associated with the body’s initial blood glucose level [22]. Meanwhile, the VFAs generated by the fermentation processes of gut microbiota can substantially promote lipid biosynthesis via the liver–gut axis [23,24]. Through in vivo stable isotope labeling and dietary interventions, it was shown that acetate is a precursor for liver synthesis of C16 and C18 fatty acids and glycerolipids [25]. The lamb live weight and growth rate are significant economic traits, and the utilization of forage represents a more effective strategy for enhancing production efficiency. It was observed that animals exhibiting elevated Kleiber ratios are regarded as efficient feed users [26,27]. Furthermore, the lamb breed, sex, season, and year were identified as the primary factors influencing growth traits and Kleiber ratios [28]. However, it remains to be determined whether intestinal VFAs, microbiota, and hepatic metabolites influence lamb body weight through the interplay of the gut–liver axis, particularly under conditions where external factors are highly consistent. Therefore, the present study was conducted to monitor the production performance, gut microbiota, VFAs, enzyme activity, tissue morphology, and liver metabolism in a model of sheep with different daily weight gains at 180 days of age with the aim of exploring the potential factors affecting the growth and development of lambs using the microbiota–gut–liver axis.

## 2. Results

### 2.1. Comparison of Dressing Percentage and Organ Indices in Lambs

Table 1 indicates that the body weight (BW) and carcass weight (CW) were significantly higher in HDAG lambs than in LADG lambs (*p* < 0.05) but significantly lower in the kidney index (*p* < 0.05), with no significant effect on the dressing percentage (DP), heart index, liver index, spleen index, and lung index (*p* > 0.05).

### 2.2. Comparison of Jejunal VFAs, Digestive Enzymes, and Morphology in Lambs

Table 2 indicates that propanoic acid (PA), butyric acid (BA), the propanoic acid ratio (PAR), the butyric acid ratio (BAR), beta-glucosidase (GLU), microcrystalline cellulase (MCC), xylanase (Xyl), carboxymethyl cellulase (CMC), and amylase (AMS) was significantly higher in HDAG lambs than in LADG lambs (*p* < 0.05) but significantly lower in acetic acid (AA), acetic acid/ propanoic acid (AA:PA), and the acetic acid ratio (AAR) (*p* < 0.05), with no significant effect on the total volatile fatty acids (TVFAs), lipase, villus height, crypt depth, and villus height/crypt depth (*p* > 0.05).

### 2.3. Analysis of the Jejunal Microbiota of HADG and LADG Lambs

#### 2.3.1. Analysis of the Microbiota Diversity of the Jejunum

**Figure 1:**
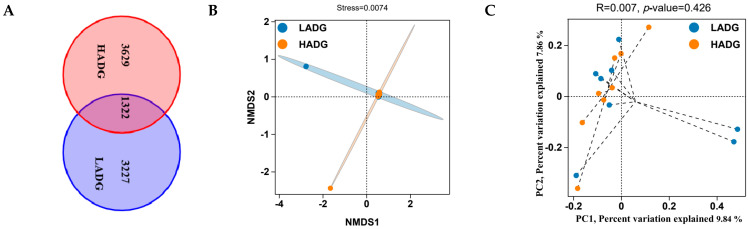
Analysis of the microbiota diversity of the jejuna of HADG and LADG lambs. (**A**) ASV Venn diagram analysis of HADG and LADG lambs. (**B**) PCoA analysis of HADG and LADG lambs. (**C**) NMDS analysis of HADG and LADG lambs.

Through Wayne diagrams, it was revealed that jejunal microbiota sequences were assigned to 8178 Amplicon Sequence Variants (ASVs), of which 1322 ASVs were shared among two groups (Figure 1A). Furthermore, based on the binary Jaccard method, the Non-Metric Multi-Dimensional Scaling (NMDS) analysis, the results show that the stress = 0.0074, indicating that the model has a certain level of reliability (Figure 1B). Meanwhile, a principal coordinates analysis (PCoA) analysis using the binary Jaccard method showed that the first and second principal components contributed 9.84% and 7.86% to sample differences, respectively. The ANOSIM results indicated no significant difference between the groups (*p* > 0.05) (Figure 1C). Table 3 indicates that ACE, Chao1, Shannon, and Simpson were not significantly different in HDAG lambs and LADG lambs (*p* > 0.05).

#### 2.3.2. Microbiota Composition and Function Prediction in the Jejunum

**Figure 2:**
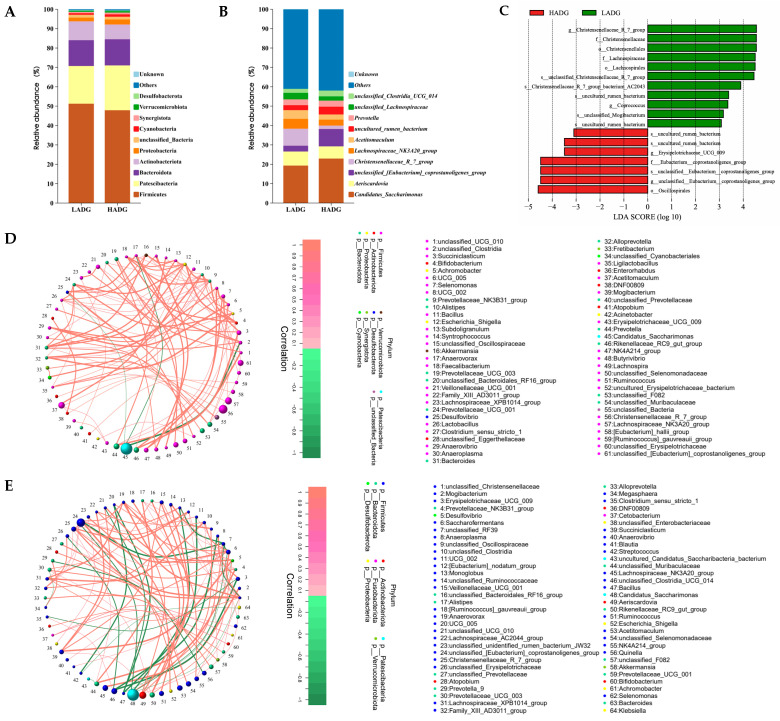

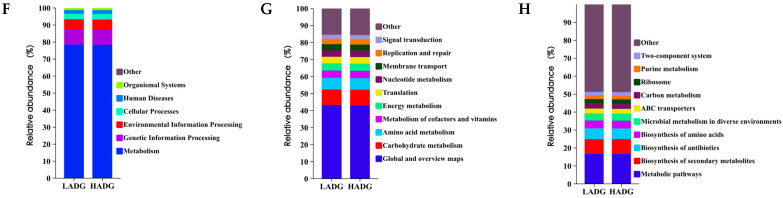
Analysis of the microbiota composition and function prediction in the jejuna of HADG and LADG lambs. HADG and LADG lamb jejunal microbiota phylum (**A**) and genus (**B**) level top 10 stacking diagrams. (**C**) LEfSe analysis jejunal biomarkers in HADG and LADG lambs. Network diagrams of LADG (**D**) and HADG (**E**) microbiota correlations at the top 80 genus level are constructed. HADG and LADG lamb jejunal microbiota functional enrichment of class 1 (**F**), class 2 (**G**), and class 3 (**H**) analyzed using PICRUSt2.

The analysis of the microbiota composition of the jejunum showed that the dominant phylum in the jejunum was Firmicutes (LADG: 51.28%; HADG: 47.90%), followed by Patescibacteria (LADG: 19.43%; HADG: 23.12%), Bacteroidota (LADG: 13.31%; HADG: 13.51%), and Actinobacteriota (LADG: 9.74%; HADG: 7.61%), with a combined percentage of more than 92.14%. However, there was no statistically significant difference in the jejunal microbiota between the HADG and LADG lambs at the top 10 phylum level (*p* > 0.05) (Figure 2A). In addition, the overall differences between the top 10 genera of jejunum were not significant, including *Candidatus_Saccharimonas* (LADG: 19.34%; HADG: 22.96%), *Aeriscardovia* (LADG: 7.24%; HADG: 6.26%), *Christensenellaceae_R_7_group* (LADG: 8.78%; HADG: 1.79%), *Lachnospiraceae_NK3A20_group* (LADG: 5.09%; HADG: 3.08%), *Acetitomaculum* (LADG: 4.49%; HADG: 2.62), *Prevotella* (LADG: 2.99%; HADG: 3.09), *unclassified_Lachnospiraceae* (LADG: 3.37%; HADG: 2.19%), and *unclassified_Clostridia_UCG_014* (LADG: 2.08%; HADG: 3.04%) (*p* > 0.05), with only *unclassified_[Eubacterium]_coprostanoligenes_group* (LADG: 3.01%; HADG: 8.97%) and *uncultured_rumen_bacterium* (LADG: 2.56%; HADG: 4.05%) being significantly different from each other (*p* < 0.05) (Figure 2B).

Additionally, the linear discriminant analysis (LDA = 3.0) effect size (LEfSe) of lamb jejunal biomarkers identified 18 significant biomarkers, of which 11 were found in LADG lambs and 7 in HADG lambs. At the genus level, LADG lambs were identified by two biomarkers: *Christensenellaceae_R_7_group* and *Coprococcus*. Meanwhile, the two identified biomarkers in HADG lambs were *Erysipelotrichaceae_UCG_009* and *unclassified_[Eubacterium]_coprostanoligenes_group* (Figure 2C). Moreover, the analysis of the top 80 genus level network diagrams depicting the microbial network in the jejuna of lambs from the LADG and HADG groups indicated significant correlations among the microbiota (*p* < 0.05) (Figure 2D,E). Notably, the jejunal microbial interactions were dominated by Firmicutes in both the LADG and HADG groups (Figure 2D,E).

Finally, Phylogenetic Investigation of Communities by Reconstruction of Unobserved States (PICRUSt2) prediction results of the LADG and HADG groups showed that the main enrichment was in the metabolic pathway and accounted for more than 78.54% and 78.40%, respectively, including the top 10 class 2 level global and overview maps (LADG: 43.36%; HADG: 43.25%), carbohydrate metabolism (LADG: 9.10%; HADG: 9.11%), amino acid metabolism (LADG: 6.79%; HADG: 6.72), metabolism of cofactors and vitamins (LADG: 4.30%; HADG: 4.27), energy metabolism (LADG: 4.17%; HADG: 4.17%), and nucleotide metabolism (LADG: 3.87%; HADG: 3.86%) (Figure 2F,G). However, HADG and LADG lamb jejunal microbiota were not significantly affected in the top 10 functional enrichment at the class 1 (Figure 2F), class 2 (Figure 2G), and class 3 levels (Figure 2H) (*p* > 0.05).

#### 2.3.3. Correlation Analysis of Jejunal Microbiota and Host Phenotypes

**Figure 3:**
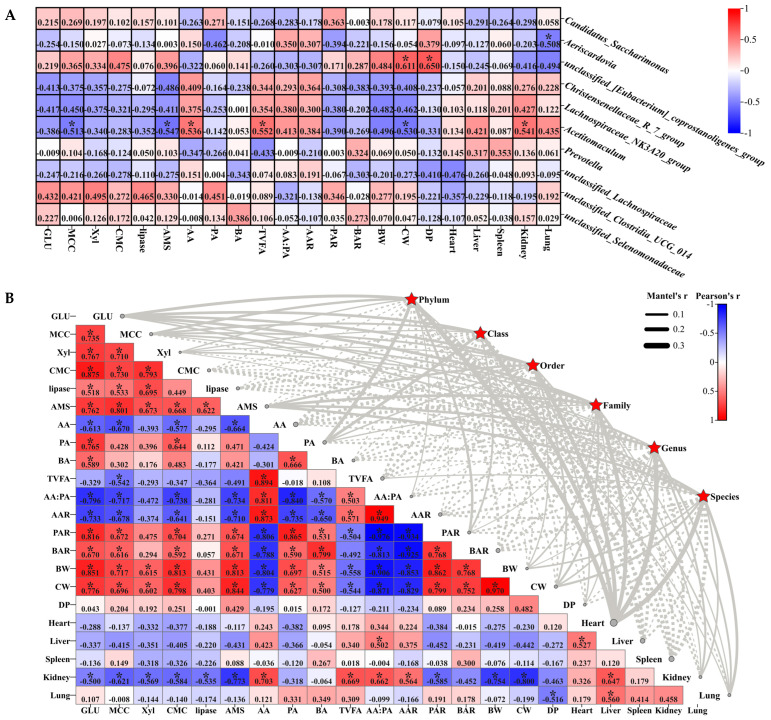
Correlation analysis of jejunal microbiota and host phenotypes. (**A**) Heat map analysis of microbiota (genus level) correlation with phenotype of HADG and LADG lambs. (**B**) Mantel’s r analysis of microbiota correlation with phenotype of HADG and LADG lambs. The edge width corresponds to the Mantel’s r statistic for the corresponding distance correlations, and the edge color indicates the significance of the Mantel’s *p* statistic, with a gray line indicating *p* > 0.05. The type of line denotes a positive (solid) or negative (dashed) correlation. Note: the values in the correlation heat map indicate the correlation coefficients. * In the correlation heat map, we indicate *p* < 0.05. Abbreviations: acetic acid (AA), propionic acid (PA), butyric acid (BA), total volatile fatty acids (TVFAs), acetic acid/propionic acid (AA:PA), acetic acid ratio (AAR), propionic acid ratio (PAR), butyric acid ratio (BAR), beta-glucosidase (GLU), microcrystalline cellulose (MCC), lipase, xylanase (Xyl), amylase (AMS), carboxymethyl cellulose (CMC), body weight (BW), carcass weight (CW), and dressing percentage (DP).

Figure 3 analyzes the correlation between the host phenotype and top 10 jejunal microbiota at the genus level (A) and for microbiota (B). Mantel’s r analysis revealed that jejunal microbiota had no significant jejunal digestive enzymes (GLU, MCC, Xyl, CMC, lipase, and AMS), VFA molar content (AA, PA, BA, and TVFAs), VFA molar ratio (AA:PA, AAR, PAR, and BAR), dressing percentage (BW, CW, and DP), and organ index effect (heart, liver, spleen, kidney, and lung indices) (*p* > 0.05) (Figure 3B). Furthermore, the jejunal digestive enzymes GLU, MCC, Xyl, CMC, lipase, and AMS were significantly positively correlated with each other (Figure 3B) (*p* < 0.05). Meanwhile, *Acetitomaculum* was significantly negative correlated with MCC and AMS (*p* < 0.05) (Figure 3A,B). Additionally, there was a significant positive correlation between AA and TVFAs as well as BA and PA in the jejunum (*p* < 0.05), while *Acetitomaculum* was significantly positively correlated with TVFAs (*p* < 0.05) (Figure 3A,B). Moreover, AA had a significant positive correlation between AA:PA and AAR, but they were all significantly negatively correlated with PAR and BAR (*p* < 0.05) (Figure 3B). Meanwhile, there was a significant positive correlation between AA:PA and AAR as well as PAR and BAR (*p* < 0.05) (Figure 3B). Additionally, CW was significantly correlated with *Acetitomaculum* (R = −0.530) and *unclassified_[Eubacterium]_coprostanoligenes_group* (R = 0.611) (*p* < 0.05) (Figure 3A). In addition, significant correlations were found between *Acetitomaculum* and the kidney index (R = 0.541), *unclassified_[Eubacterium]_coprostanoligenes_group* and DP (R = 0.650), and *Aeriscardovia* and the lung (R = −0.50) (*p* < 0.05) (Figure 3A). However, the kidney was significantly negatively correlated with GLU, MCC, Xyl, CMC, lipase, AMS, PAR, BW, and CC (*p* < 0.05), but it was significantly positively correlated with AA, TVFAs, AA:PA, and AAR (*p* < 0.05) (Figure 3B). Moreover, the liver showed a significant positive correlation with the heart, lung, and kidney (*p* < 0.05), while DP and the lung had a significant negative correlation (*p* < 0.05) (Figure 3B). Notably, BW and CW were significantly positively correlated with GLU, MCC, Xyl, CMC, AMS, PA, BA, PAR, and BAR (*p* < 0.05), but they demonstrated significant negative correlations with AA, TVFAs, AA:PA, and AA (*p* < 0.05) (Figure 3B).

### 2.4. Analysis of the Liver Metabolism of HADG and LADG Lambs

#### 2.4.1. Overall Analysis of Liver Metabolites

**Figure 4:**
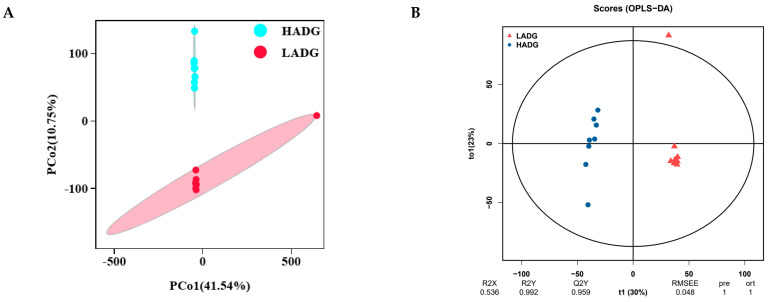

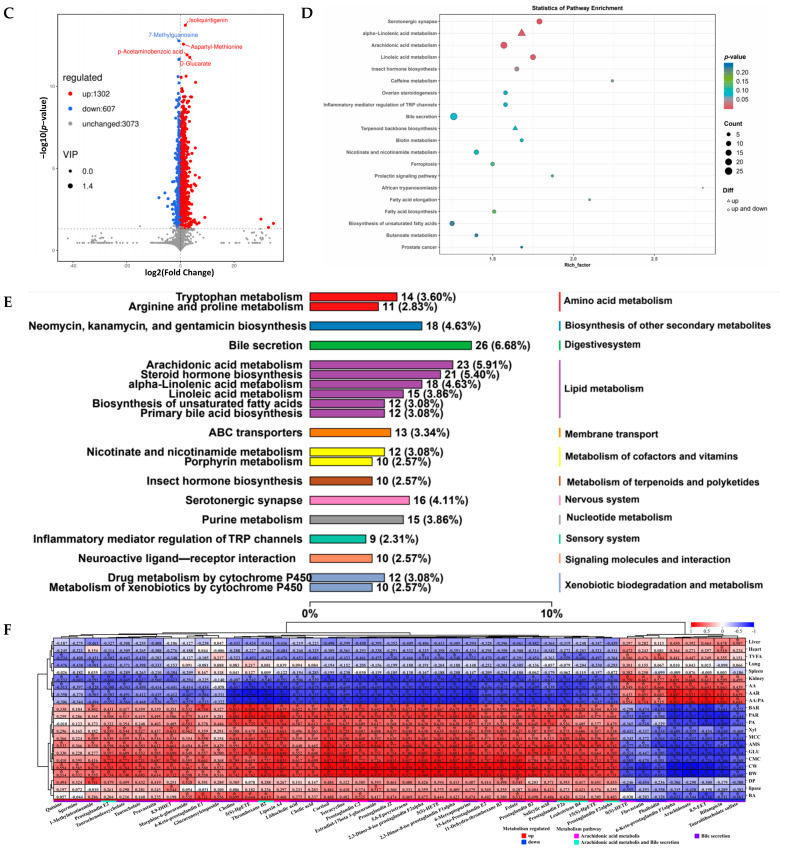
Overall analysis of liver metabolites of HADG and LADG lambs. (**A**) Liver metabolism PCoA analysis of HADG and LADG lambs. (**B**) Plotting of OPLS-DA model scores of LADG and HADG lambs. (**C**) Plotting of volcano diagram of LADG vs. HADG lambs. KEGG enrichment sites (**D**) and classification map (**E**) of liver top 20 differential metabolites. The different colored entries in the figure represent the hierarchical classification of KEGG pathway annotations, corresponding to KO pathway level 2 and the KEGG pathway name. Additionally, the lengths and values of the bars indicate the number and proportion of differential metabolites associated with that pathway. (**F**) Correlation analysis between HADG and LADG lamb phenotypes and the liver differential enrichment metabolites from the two most abundant pathways. The values in the correlation heat map indicate the correlation coefficients. * In the correlation heat map, we indicate *p* < 0.05. Abbreviations: acetic acid (AA), propionic acid (PA), butyric acid (BA), total volatile fatty acids (TVFAs), acetic acid/propionic acid (AA:PA), acetic acid ratio (AAR), propionic acid ratio (PAR), butyric acid ratio (BAR), beta-glucosidase (GLU), microcrystalline cellulose (MCC), lipase, xylanase (Xyl), amylase (AMS), carboxymethyl cellulose (CMC), body weight (BW), carcass weight (CW), and dressing percentage (DP).

It can be seen from PCoA (Figure 4A) and orthogonal projections to latent structure discriminate analysis (OPLS-DA) (Figure 4B) that the samples of each group were distinguishable. In this case, the model is reliable (Q2Y = 0.959 > 0.50) and can be used to screen for differential metabolites. Next, volcano plots were created according to VIP > 1 and *p* < 0.05, in which 4982 metabolites were identified, containing 1909 (1302 up-regulated and 607 down-regulated) differential metabolites (Figure 4C).

Furthermore, liver differential metabolites were annotated using the Kyoto Encyclopedia of Genes and Genomes (KEGG) database, and the top 20 pathways with the highest number of selected metabolites are shown in Figure 4D,E, including amino acid metabolism (tryptophan metabolism and arginine and proline metabolisms), biosynthesis of other secondary metabolites (neomycin, kanamycin, and gentamicin biosynthesis), digestive system (bile secretion), lipid metabolism (arachidonic acid metabolism, steroid hormone biosynthesis, alpha-Linolenic acid metabolism, linoleic acid metabolism, biosynthesis of unsaturated fatty acids, and primary bile acid biosynthesis), membrane transport (ABC transporters), metabolism of cofactors and vitamins (nicotinate and nicotinamide metabolisms and porphyrin metabolism), metabolism of terpenoids and polyketide (insect hormone biosynthesis), nervous system (serotonergic synapse), nucleotide metabolism (purine), sensory system (inflammatory mediator regulation of TRP channels), signaling molecules and interaction (neuroactive ligand–receptor interaction), and xenobiotics biodegradation and metabolism (metabolism of xenobiotics by cytochrome P450 and drug metabolism via cytochrome P450). In addition, the two most abundant pathways for liver differential metabolite enrichment were bile secretion and arachidonic acid metabolism (Figure 4E).

Subsequently, in Figure 4F, we analyzed the correlation between bile secretion and arachidonic acid metabolic pathways of the liver’s most enriched differential metabolites and host phenotypes. Overall, we found that BAR, PAR, PA, Xyl, MCC, AMS, GLU, CMC, CW, and BW were positively correlated with up-regulated metabolites and negatively correlated with down-regulated metabolites in HADG lambs compared to LADG lambs, as shown in Figure 4F. However, the kidney, AA, AA:PA, and AAR were negatively correlated with up-regulated metabolites and positively correlated with down-regulated metabolites in HADG lambs compared to LADG lambs, as shown in Figure 4F.

#### 2.4.2. Liver Differential Metabolite KEGG Enrichment and Phenotype Correlation Analysis

**Figure 5:**
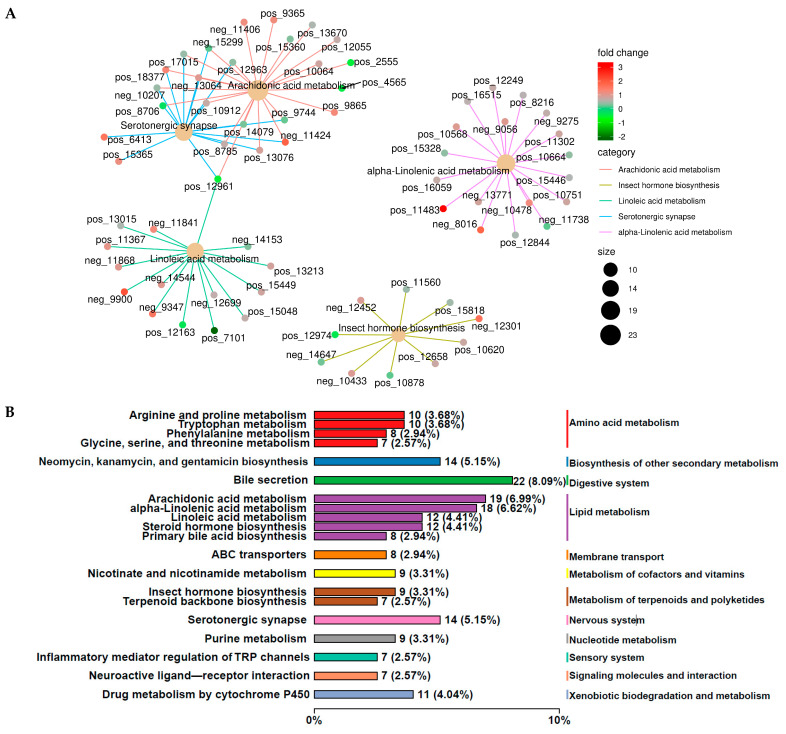

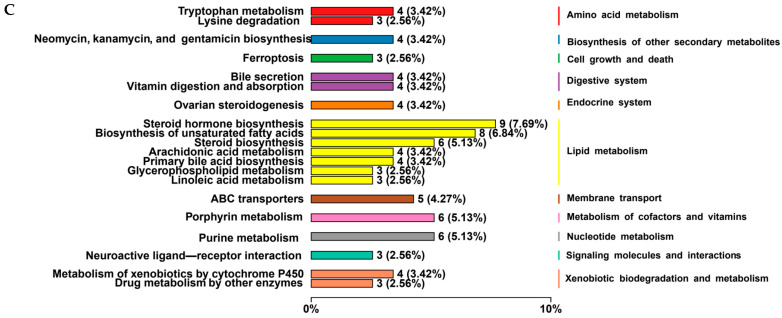
Liver differential metabolite KEGG enrichment and host phenotype correlation analysis of HADG and LADG lambs. (**A**) Network map of differential metabolites in the liver KEGG enriched top five pathways. Moreover, the size of the yellowish nodes in the graph corresponds to the quantity of enriched differential metabolites, while the smaller nodes connected to them represent the specific metabolites that have been annotated to the pathway, and the change in color indicates that the fold of differences takes the value of log2. Figure note size represents the number of enriched different metabolite. The categorization of the top 20 KEGG pathways that up-regulate (**B**) and down-regulate (**C**) liver metabolites by HADG compared to LADG. The different colored entries in the figure represent the hierarchical classification of KEGG pathway annotations, corresponding to KO pathway level 2 and the KEGG pathway name. Additionally, the lengths and values of the bars indicate the number and proportion of differential metabolites associated with that pathway.

The KEGG top five differential metabolites are predominantly enriched in serotonergic synapse, alpha-Linolenic acid metabolism, arachidonic acid metabolism, arachidonic acid metabolism, and insect hormone biosynthesis (*p* < 0.05) (Figure 5A). Meanwhile, we heat mapped the correlation between the enrichment of liver top five metabolic pathways of differential metabolites and microbiota markers (Supplementary Figure S1) and lamb phenotype (Supplementary Figure S2). Overall, our analysis found that GLU, MCC, Xyl, AMS, PA, BA, PAR, BAR, BW, *Erysipelotrichaceae_UCG_009,* and *unclassified_[Eubacterium]_coprostanoligenes_group* were positively correlated with up-regulated metabolites and negatively correlated with down-regulated metabolites in HADG lambs (*p* < 0.05) (Supplementary Figures S1 and S2). However, AA, TVFAs, AA:PA, AAR, *Christensenellaceae_R_7_group*, and *Coprococcus* were negatively correlated with up-regulated metabolites and positively correlated with down-regulated metabolites in HADG lambs (*p* < 0.05) (Supplementary Figures S1 and S2).

Additionally, HADG lamb liver up-regulated differential metabolites were primarily enriched in the amino acid metabolism and lipid metabolism (*p* < 0.05) (Figure 5B). In contrast, the up-regulated differential metabolites in LADG lamb livers were predominantly enriched in lipid metabolism (*p* < 0.05) (Figure 5C). Furthermore, our found that BAR, PA, Xyl, MCC, PAR, GLU, AMS, CW, BW, and CMC were positively correlated with up-regulated metabolites (such as spermine, cholic acid, succinic acid, creatinine, agmatine, rumenic acid, choloyl-CoA, betaine, Choline, etc.) and negatively correlated with down-regulated metabolites (such as Hexadecanoic acid, 7alpha-Hydroxycholest-4-en-3-one, 16alpha-Hydroxyestrone, Calcitriol, etc.) in HADG lambs compared to LADG lambs (Supplementary Figure S3). However, the kidney, AA, AA:PA, and AAR were negatively correlated with up-regulated metabolites and positively correlated with down-regulated metabolites in HADG lambs compared to LADG lambs (*p* < 0.05) (Supplementary Figure S3).

### 2.5. Liver Metabolite and Jejunal Microbiota Correlation Analysis

Liver metabolite modules and jejunal microbiota were screened for the top 30 based on a CCP < 0.05. Data with at least one correlation coefficient in this range were used for metabolite module–microbiota correlation plots and network diagrams, which containing 12 metabolic modules and 12 phyla (Supplementary Tables S1 and S2). Overall, the correlation between microbiota and metabolic modules was |CC|> 0.5 (Supplementary Table S2). Furthermore, we conducted an analysis of the correlation between metabolic modules and microorganisms, utilizing a threshold of |CC| > 0.8 and CCP < 0.05. The results show that Dadabacteria shows a significant negative correlation with MEbrown and MEred, but it shows a significant positive correlation with MEblue (*p* < 0.05) (Supplementary Figures S4 and S5). Meanwhile, Deinococcota shows a significant negative correlation with MEgreenyellow, but it shows a significant positive correlation with MEblack (*p* < 0.05) (Supplementary Figures S4 and S5).

**Figure 6:**
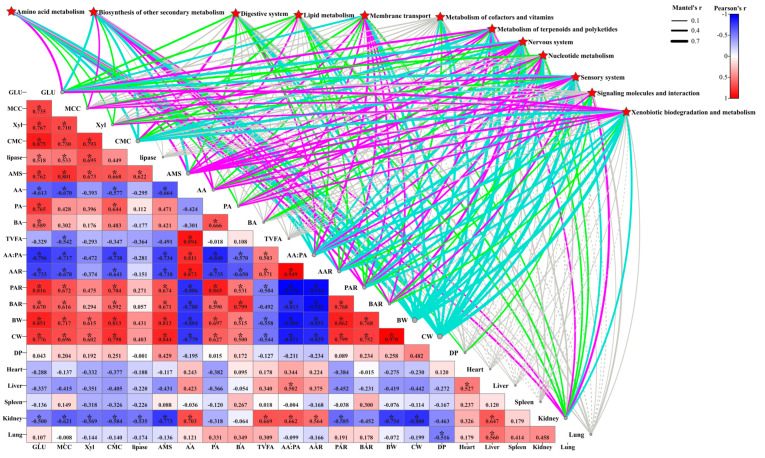
Mantel’s r analysis of the liver KEGG enrichment pathway in correlation with the phenotype of HADG and LADG lambs. The edge width corresponds to the Mantel’s r statistic for the corresponding distance correlations, and the edge color indicates the significance of the Mantel’s *p* statistic, with a turquoise line indicating *p* ≤ 0.001, magenta line indicating 0.001 < *p* ≤ 0.01, green line indicating 0.01 < *p* ≤ 0.05, and gray line indicating *p* > 0.05. The type of line denotes a positive (solid) or negative (dashed) correlation. Note: the values in the correlation heat map indicate the correlation coefficients. * In the correlation heat map, we indicate *p* < 0.05. Abbreviations: acetic acid (AA), propionic acid (PA), butyric acid (BA), total volatile fatty acids (TVFAs), acetic acid/propionic acid (AA:PA), acetic acid ratio (AAR), propionic acid ratio (PAR), butyric acid ratio (BAR), beta-glucosidase (GLU), microcrystalline cellulose (MCC), lipase, xylanase (Xyl), amylase (AMS), carboxymethyl cellulose (CMC), body weight (BW), carcass weight (CW), and dressing percentage (DP).

The results of plotting correlation chord plots and network diagrams by keeping the correlation results of the top 30 frequencies of differential metabolites/differential microbiota (|CC| > 0.8 and CCP < 0.05) containing at least one set of correlation coefficients with an absolute value in the top 30 showed that *Candidatus_Actinomarina*, *Candidatus_Puniceispirillum*, *Christensenellaceae_R_7_group*, *Clostridium_sensu_stricto_8*, *Garicola*, *Ochrobactrum*, *Pseudoalteromonas*, *unclassified_AEGEAN_169_marine_group,* and Licoagrodin were positively correlated, but they were negatively correlated with all other metabolites (such as valeroyl salicylate, C20912, hexadecanedioate, diamsar chelate, guanosine monophosphate, dTDP-L-epivancosamine, methylitaconate, p-Tolyl Sulfate, butanamide, etc.) (Supplementary Figures S6 and S7). Notably, Mantel’s r analysis showed that the liver KEGG enrichment pathway was significantly correlated with lamb jejunal digestive enzymes (GLU, MCC, Xyl, CMC, and AMS), VFA molar content (AA, PA, and BA), VFA molar ratio (AA:PA, AAR, PAR, and BAR), dressing percentage (BW, CW, and DP), and organ indices (kidney index) but not significantly correlated with lipase, TVFAs, the heart index, liver index, spleen index, and lung index (*p* < 0.05) (Figure 6).

## 3. Discussion

### 3.1. Effects of Different Growth and Development Levels on Slaughter Performance and Jejunal Digestion and Tissue Morphology in Lambs

Animal growth is defined by the increase in weight and volume of body tissues and the accumulation of components like protein, lipids, water, and ash [29]. Of note, body weight is a key economic factor in sheep production, being affected by genetic and environmental interactions, including host genetics and gastrointestinal tract microecology [14,30]. Moreover, nutrients, microbiota, and metabolites interact with each other in the gut–liver cycle to regulate gut and liver functions [12]. The research has indicated a strong correlation between rumen microbiota and both rumen fermentation and growth performance in young goats [9]. Furthermore, the optimal growth and development of young ruminants play a crucial role in the establishment of their physiological functions, which, in turn, affect their productive and reproductive performances, as well as their overall health status in adulthood [7,8]. Thus, we hypothesized that changes in weight gain in lambs may be linked to gut microbiota, volatile fatty acids, digestive enzymes, and body metabolism [14,29,31]. The study found that HADG lambs had significantly increased body weight, carcass weight, propanoic acid, butyric acid, propanoic acid ratio, butyric acid ratio, beta-glucosidase, microcrystalline cellulase, and carboxymethyl cellulase. However, it significantly decreased the kidney index, acetic acid, acetic acid/propanoic acid, and acetic acid ratio. Research has demonstrated that propionic acid stimulates the expression of essential genes involved in gluconeogenesis via the gut–brain axis, thereby enhancing glucose metabolism and maintaining energy homeostasis in the small intestine [32,33]. Furthermore, augmenting propionic acid production through nutritional interventions has the potential to elevate hepatic gluconeogenesis, consequently leading to the improved production performance of ruminants [34]. Moreover, butyric acid can improve the ability of intestinal absorption of nutrients [35,36] and improve ruminant performance [37,38]. Though correlation analysis, we found that body weight was found to be significantly positively correlated with the VFA molar ratio and content (propionic and butyric acids) of jejunal, while it was significantly negatively correlated with acetic acid, TVFAs, acetic acid molar ratio, and acetic acid/propanoic acid. This result is consistent with that of Wang et al., who found that the ADG of 6-month-old goats positively correlated with the rumen fluid propionic and butyric acid concentrations and propionic acid molar ratio while negatively correlating with the acetic acid molar ratio and acetic acid/propanoic acid [9]. This result may be related to the fact that propionic acid and butyric acid contents are higher in efficient animals [39]. The potential for integrating nutritional interventions, such as grain feed [40] and monensin [41,42], aimed at enhancing the levels and ratios of propionic and butyric acids while simultaneously reducing the acetic acid-to-propionic acid ratio, to promote gastrointestinal fermentation in fattening lambs and subsequently influence their performance requires further elucidation. Additionally, beta-glucosidase, microcrystalline cellulase, carboxymethyl cellulase, and amylase exhibited significant positive correlations with the ratios of propionic and butyric acids while demonstrating significant negative correlations with the molar ratio of acetic acid and the acetic acid/propanoic acid ratio. The gut microbiota is considered a metabolic “organ” that not only contributes to obtaining nutrients and energy from ingested food but also produces many metabolites that regulate host metabolism [12], such as VFAs, bridge microbiota, and promote host interactions, influencing metabolism, immune responses, and gut microbiota structure [14,15]. Microbiota, known as the “second genome”, can directly or indirectly affect the animal’s growth development and body composition [9,14,29,43,44]. Consequently, we hypothesized that the alterations in jejunal VFAs and digestive enzymes may be associated with the microbiota [9,15,45]. Subsequently, we analyzed the jejunal microbiota of lambs of different growth and development levels.

### 3.2. Effects of Different Growth and Development Levels on Jejunal Microbiota in Lambs

The gastrointestinal microbiota is strongly associated with host traits in integrating environmental signals into host responses [14,46], such as influencing feed efficiency [6,47] and body weight [14]. Therefore, we analyzed the jejunal microbiota using different growth development lambs. The study demonstrated that higher microbiota diversity in lambs may improve their adaptability to environmental changes and sustain internal homeostasis [48]. The experiment found no significant differences in jejunal microbiota diversity among lambs of different growth and development levels. This may be attributed to the susceptibility of animal gastrointestinal microbiota communities to factors such as host genetics, age, sex, diet, and geographic range [14,49,50], whereas different growth and development levels do not have a significant effect on microbiota diversity. Further results indicated that the jejunal microbiota composition at the level of the top 10 phyla and genera was not significantly influenced in different growth rates of lambs and with the dominant phylum Firmicutes. This result is consistent with the observation that the relative abundance of Firmicutes is higher in the jejunum of sheep [51]. Furthermore, Firmicutes was identified as the predominant microbiota phylum. Moreover, this was found by constructing a network diagram of genus-level communities with top 80 abundance, which was dominated by the phylum Firmicutes in both HADG and LADF lambs. This result supports previous findings that Firmicutes microbiota interactions are more pronounced [30,52], and Firmicutes encoded enzyme genes related to energy metabolism [53]. Additionally, the dominant microbiota at the genus level of the jejunum in lambs was *Candidatus_Saccharimonas*. The study demonstrates that *Candidatus-Saccharimonas*, a bacterium endemic to the foregut segment of sheep, is mainly involved in amino acid biosynthesis, the metabolism of energy substrates, and the maintenance of gut function, such as anti-inflammation, and contributes to IL-4 and IL-10 secretion [9,54,55,56]. Furthermore, the functional enrichment of the top 10 microbiota in the jejuna of lambs exhibiting varying growth rates was not significantly influenced at the class 1, class 2, and class 3 levels, and functional enrichment was predominantly found in the metabolic pathway. This finding is consistent with previous studies that identified microbiota functional enrichment metabolic pathways [57]. Our analysis of microbiota and jejunal fermentation parameters at the top 10 genus level revealed significant positive correlations between *Acetitomaculum* and both acetic acid and TVFAs. Furthermore, *Acetitomaculum* is another well-known acetate- and lactic acid-producing bacterium [58]. However, other microbiota at the genus level (such as *Candidatus_Saccharimonas*, *Aeriscardovia*, *Christensenellaceae_R_7_group*, etc.) within the top ten did not exhibit a significant effect on VFAs in individuals with jejunal chyme. It is worth noting that there are intricate interactions among microbiota that could influence their relationship with phenotypes [59,60]. Mantel’s r analyzed revealed that jejunal microbiota had no significant correlation jejunal VFA molar content (AA, PA, BA, and TVFAs), VFA molar ratio (AA:PA, AAR, PAR, and BAR), which is consistent with the results of previous studies that found microbiota correlated with VFAs in sheep [52]. Furthermore, there exist intricate interactions among microbiota, and Mantel’s r primarily focuses on linear correlations within the matrix, thereby neglecting non-linear relationships that could influence the correlation [14]. While dominant bacteria are vital for host physiology, low-abundance bacteria deserve attention due to their greater taxonomic diversity and potential roles in host functions. In complex rumen ecosystems, microbiota interactions may be more important for ecosystem function than abundance [9,61]. The enterohepatic axis theory suggests that gut microbiota impacts host metabolism, with changes in hepatic metabolites reflecting gut microbiota metabolism [12]. Thus, significant differences between jejunal VFA molar content and ratios in lambs with different growth rates, which may play a crucial role in targeting and regulating liver metabolism [16].

### 3.3. Effects of Different Growth and Development Levels on Liver Metabolites in Lambs

The microbiota and host metabolism play crucial roles in the regulation of nutritional homeostasis within the host. For instance, when members of the intestinal flora effectively utilize a substantial proportion of intestinal amino acids, this process influences the uptake and utilization of amino acids by host tissues and organs, such as the intestines and liver [60]. The KEGG enrichment analysis of liver different metabolites were mainly enriched in amino acid metabolism, digestive system, and lipid metabolism, especially the digestive system (bile secretion) and lipid metabolism (arachidonic acid metabolism) pathways. Meanwhile, we found that beta-glucosidase, microcrystalline cellulase, xylanase, amylase, propanoic acid, butyric acid, the propanoic acid ratio, butyric acid ratio, body weight, *Erysipelotrichaceae_UCG_009,* and *unclassified_[Eubacterium]_coprostanoligenes_group* were positively correlated with up-regulated and down-regulated metabolites in the top five differential metabolic pathways (serotonergic synapse, alpha-Linolenic acid metabolism, arachidonic acid metabolism, arachidonic acid metabolism, and insect hormone biosynthesis). However, acetic acid, TVFAs, acetic acid/ propanoic acid, acetic acid ratio, *Christensenellaceae_R_7_group*, and *Coprococcus* were negatively correlated with the up-regulated metabolites and positively correlated with the down-regulated top five differential metabolic pathways metabolites in lambs. This finding further indicates that the gut–liver axis is instrumental in maintaining homeostasis within the gastrointestinal system and in the metabolism of carbohydrates, lipids, fatty acids, and amino acids [10,11,12,24]. Next, Mantel’s r analysis showed that the liver KEGG enrichment pathway was significantly correlated with lamb jejunal digestive enzymes (beta-glucosidase, microcrystalline cellulase, xylanase, carboxymethyl cellulase, and amylase), VFA molar content (acetic acid, propanoic acid, and butyric acid), VFA molar ratio (acetic acid/ propanoic acid, acetic acid ratio, propanoic acid ratio, and butyric acid ratio), dressing percentage (body weight, carcass weight, and dressing percentage), and organ indices (kidney index). In addition, there was a significant correlation between gut microbiota and hepatic metabolism. Thus, gut-produced VFAs and the hepatic bile secretion pathway play a role in maintaining homeostasis within the gastrointestinal system through the gut–liver axis, which may influence lamb growth by regulating lipid and amino acid metabolisms in animals [10,11,12,13,16,23,24,25].

Furthermore, our further analysis revealed that HADG lamb liver up-regulated differential metabolites were mainly enriched in amino acid metabolic pathways (arginine and proline; tryptophan; phenylalanine; and glycine, serine, and threonine metabolisms) and lipid metabolism (arachidonic acid, alpha-Linolenic acid, linoleic acid, steroid hormone biosynthesis, and primary bile acid biosynthesis). The research provides evidence that amino acid metabolism (such as glycine, serine, and threonine metabolism, tyrosine metabolism, arginine and proline metabolism, cysteine and methionine metabolism, and phenylalanine metabolism) is essential for forming metabolic precursors, regulating meat quality in sheep, and facilitating epigenetic modification [62,63]. Moreover, glycine, serine, and threonine metabolic pathways are thought to provide the main energy metabolism precursor substance for the TCA cycle [64]. Meanwhile, propionic acid participates in the TCA cycle as an energy intermediate, providing the body with the required energy. This result may be related to the fact that animals with high Kleiber ratios have higher feed efficiency, and efficient animals have higher propionic and butyric acid contents [26,27,39]. Meanwhile, lipids are fundamental metabolites that serve multiple functions, including acting as energy sources, structural components, and signaling mediators. Additionally, lipid metabolites are crucial as signaling molecules in the regulation of energy and immune homeostasis [65]. Interestingly, phenylalanine metabolism and purine metabolism were specific to the microbiota community [66]. Thus, the observed enrichment of phenylalanine and purine metabolism pathways in lambs may be linked to their gastrointestinal microbiota structure [66], but further clarification is needed. Overall, our analysis revealed that HADG lambs compared to LADG lambs exhibited an up-regulation of metabolites (such as succinic acid, rumenic acid, betaine, cortisol, betaine aldehyde, choline, L-Tyrosine, L-cysteine, O-phospho-L-serine, L-arginine phosphate, OPC4-CoA, D-Proline, etc.) that were positively correlated with the butyric acid ratio, propanoic acid, xylanase, microcrystalline cellulase, propanoic acid ratio, beta-glucosidase, amylase, carcass weight, body weight, and carboxymethyl cellulase, while these metabolites were negatively correlated with the kidney, acetic acid, acetic acid/ propanoic acid, and acetic acid ratio. For example, succinate is an intermediate metabolite in the Krebs cycle, being involved in inflammatory responses, skeletal muscle protein synthesis, myofibrillar restructuring, energy supply, and glucose homeostasis [67,68], while it also acts as an epigenetic regulator in gene transcription, translation, and post-translational modification [69]. It has been demonstrated that succinic acid is a substrate of the intestinal gluconeogenesis pathway and improves glucose homeostasis in the body, while propionic and butyric acids also play significant roles in this process [70]. Additionally, succinic acid has been shown to markedly improve growth performance in animals [30,71,72,73]. Betaine enhances growth performance by providing methyl groups for the epigenetic regulation of liver genes and is essential for maintaining osmotic pressure, regulating protein function, and improving lipid metabolism [74,75,76]. In addition, studies have demonstrated that supplementing animal feed with betaine can significantly enhance growth performance [77,78,79]. This finding indicates that small molecule compounds, including VFAs, bile acids, succinic acid, betaine, and others, which are transported through the liver–gut axis during microbiota–host interactions, are essential signaling molecules that influence animal growth and development, lipid metabolism, and energy expenditure [14,15,16,17,19,23,24,76]. In summary, the liver–gut axis can be adapted to animal growth and development by modulating changes in metabolite enrichment and altering parameters of gut fermentation.

## 4. Materials and Methods

### 4.1. Lambs and Experimental Design

With reference to the Kleiber ratio [26,27,28], 100 Hu lambs (males) of similar birth weight and age were selected from the Science and Technology Service Workstation of Jiangxi Academy of Agricultural Sciences (Ganzhou Lvlinwan Agriculture and Animal Husbandry Co., Ltd., Ganzhou, China). The lambs were weaned at 45 days and raised under highly consistent feeding and environment conditions until 180 days of age. All animal experimental designs and feeding management were approved by the Institute of Animal Husbandry and Veterinary, Jiangxi Academy of Agricultural Sciences (2010-JAAS-XM-01, Nanchang, China). Throughout the study, all lambs had consistent feeding conditions with ad libitum access to feed and water, receiving a total mixed diet at 08:30 and 17:30 daily. At 180 days of age, based on the Kleiber ratio, we selected the 8 Hu lambs with the highest mean daily gain (HADG) and the 8 Hu lambs with the lowest mean daily gain (LADG) from 100 male lambs for slaughter and evaluated relevant parameters. Slaughter procedures were performed according to operating procedures of livestock and poultry slaughtering for sheep and goats (NY/T 3469-2019, Ministry of Agriculture, China).

### 4.2. Sample Collection and Processing

After the lambs were slaughter, the jejunal chyme immediately was collected in sterile tubes, stored at −80 °C for VFAs, enzyme activity, and 16S rRNA sequencing analysis. Meanwhile, jejunal tissues were also fixed for the villus height and crypt depth measurements. Furthermore, liver samples were immediately collected from the same locus of the liver and then quickly frozen in liquid nitrogen and transported to the laboratory for storage at −80 °C until liver metabolomics analysis.

### 4.3. Analysis of Jejunal VFA, Digestive Enzymes, and Morphology

The molar concentration of VFAs (AA, PA, and BA) in the jejunum was quantified using gas chromatography (GC-7890B, Agilent Technologies, Petaling Jaya, Malaysia) through an internal standard (2-ethylbutyric acid) method, and the specific method used referred to the study of [57]. Subsequently, the TVFAs, AA:PA, and VFA molar proportion (AAR, PAR, and BAR) were calculated. Meanwhile, we evaluated the digestive enzymes (GlU, MCC, lipase, Xyl, AAMS, and CMC) in chyme with reference to the detailed instructions of the kit (Shanghai Kexing Biotechnology Co., Ltd., Shanghai, China). Furthermore, a tissue block measuring approximately 1 cm^2^ of jejunum was obtained, gently rinsed with saline, and subsequently fixed in a paraformaldehyde solution. Following adequate fixation, the tissue was stained with hematoxylin and eosin, and sections were prepared for analysis. The height of the villi, measured from the tip of the villi to the villus–crypt junction, and the depth of the crypts, measured from the villus–crypt junction to the base of the crypts [80], were assessed using an Eclipse Ci-L photomicroscope (Nikon, Tokyo, Japan) in conjunction with Image-Pro Plus 6.0 image processing software (Media Cybernetics, Inc., Rockville, MD, USA). The ratio of villus height to crypt depth was then calculated.

### 4.4. DNA Extraction and Analysis of Bacterial Community in Jejuna

Bacterial DNA was extracted from 16 jejunal chyme samples using the TGuide S96 Magnetic Stool DNA Kit (Tiangen Biotech (Beijing) Co., Ltd., Beijing, China). The hypervariable region V3–V4 of the bacterial 16S rRNA gene was amplified with primer pairs 338F: 5′-ACTCCTACGGGAGGCAGCA-3′ and 806R: 5′-GGACTACHVGGGTWTCTAAT-3′. The PCR amplification products were subsequently purified utilizing a purification kit (Omega Inc., Norcross, GA, USA), quantified with the Qsep-400 (BiOptic, Inc., New Taipei City, Taiwan, China), and paired-end sequenced (2 × 250 bp) using an Illumina novaseq6000 (Beijing Biomarker Technologies Co., Ltd., Beijing, China). The raw data obtained from sequencing were subjected to filtering using Trimmomatic (version 0.33) [81]. Subsequently, the primer sequences were identified and removed utilizing Cutadapt (version 1.9.1) [82]. Next, USEARCH (version 10) [83] was employed to splice the double-ended reads and eliminate chimeras (UCHIME [84], version 8.1), thereby yielding high-quality analyzed sequences. Finally, high-quality data were denoised with the DADA2 [85] method in QIIME2 (version 2020.6) [86], and ASVs were classified using the Naive Bayes classifier based on the SILVA database (Release138, http://www.arb-silva.de, accessed on 4 April 2023) [87], with a 70% confidence threshold.

### 4.5. Metabolome Sequencing and Bioinformatics Analysis

Metabolite assays are described in detail with reference to the previous article by Wang et al. [30]. Briefly, 50 mg of liver tissue was mixed with 1000 μL of an extraction solution containing an internal standard. After vortexing and adding magnetic beads for grinding, the sample was sonicated. Following standing and centrifugation, the supernatant was collected for vacuum drying. Next, a liquid mass spectrometry system (Waters Acquity I-Class PLUS ultra-high performance liquid tandem Waters Xevo G2-XS QT high-resolution mass spectrometer, Waters, Milford, MA, USA) equipped with MassLynx V4.2 (Waters, Milford, MA, USA) data acquisition software was utilized, while Progenesis QI software (version 4.0) [88] facilitated peak extraction and alignment comparison to identify the metabolites for further analysis. Finally, the identified metabolites were annotated using the KEGG (https://www.genome.jp/kegg/, accessed on 4 March 2024) database, and the annotated metabolites were then mapped to the KEGG pathway database [89,90].

### 4.6. Data Statistics and Analysis

The dressing percentage (BW, CW, and DP), organ indices (heart, liver, spleen, kidney, and lung indexes), jejunal chyme VFA molar content (AA, PA, BA, and TVFA), VFA molar ratio (AA:PA, AAR, PAR, and BAR), digestive enzymes (GLU, MCC, Xyl, CMC, lipase, and AMS), and morphology (villus height, crypt depth, and villus height/crypt depth) were determined using the SPSS normality test followed by an independent sample *t*-test, and the data were analyzed using SPSS software (version 26.0) (SPSS Inc., Chicago, IL, USA). The data were represented as the means ± SEMs. *p* < 0.05 was considered significant. The alpha diversity index (ACE, Chao1, Simpson, and Shannon) of jejunal samples was assessed with QIIME2 [86]. The beta diversity of microbiota communities was analyzed using binary Jaccard indices with principal coordinates analysis (PCoA [91]) and Non-Metric Multi-Dimensional Scaling (NMDS [92]). Linear discriminant analysis (LDA = 3.0) effect size (LEfSe [93]) was used to find statistically different biomarkers. In addition, correlation network maps were constructed with the abundance top 80, *p* < 0.05, at the genus levels. Furthermore, sequence functional abundance was predicted using PICRUSt2 [94] (v2.2.0-b). Next, orthogonal projections to latent structures discriminant analysis (OPLS-DA [95], version 1.6.2) was employed to identify metabolic differences between the two groups. Finally, microbiota and metabolite data with a metabolite and microbiota correlation CCP < 0.05 and frequency counts in the top 30 were selected for correlation diagrams. Then, data with |CC| > 0.8 and CCP < 0.05 were filtered to identify metabolites and microbiota among the top 30, retaining at least one set of absolute correlation coefficients [96].

## 5. Conclusions

The study indicates that there were no significant differences in the jejunal microbiota alpha diversity or in the dominant microbiota compositions and functions of different average daily weight gain lambs. However, the body weight, carcass weight, VFA molar content (propanoic acid and butyric acid), VFA molar ratio (propanoic acid ratio and butyric acid ratio), and digestive enzymes (beta-glucosidase, microcrystalline cellulase, xylanase, and carboxymethyl cellulase) were significantly higher in HDAG lambs than in LADG lambs but significantly lower in the kidney index, VFA molar content (acetic acid), and VFA molar ratio (acetic acid/ propanoic acid and acetic acid ratio). Furthermore, HADG lamb liver up-regulated differential metabolites were mainly enriched in amino acid metabolic pathways (arginine and proline; tryptophan; phenylalanine; and glycine, serine, and threonine metabolisms) and lipid metabolism (arachidonic acid, alpha-Linolenic acid, linoleic acid, steroid hormone biosynthesis, and primary bile acid biosynthesis). Overall, our analysis revealed that HADG lambs compared to LADG lambs exhibited an up-regulation of metabolites (such as spermine, cholic acid, 2-Aminomuconate semialdehyde, succinic acid, creatinine, rumenic acid, choloyl-CoA, betaine, cortisol, betaine aldehyde, choline, gamma-L-Glutamylputrescine, L-Tryptophan, alpha-Linolenic acid, L-Tyrosine, L-cysteine, O-Phospho-L-serine, L-Arginine phosphate, OPC4-CoA, (R)-(Indol-3-yl)lactate, D-Proline, Androsterone, etc.) that were positively correlated with the butyric acid ratio, propanoic acid, xylanase, microcrystalline cellulase, propanoic acid ratio, beta-glucosidase, amylase, carcass weight, body weight, and carboxymethyl cellulase, while these metabolites were negatively correlated with the kidney, acetic acid, acetic acid/ propanoic acid, and acetic acid ratio. Furthermore, there was a significant correlation between liver metabolism and jejunal microbiota. It is evident that the liver–gut axis plays a significant role in regulating animal growth and development. Furthermore, the liver–gut axis can be adapted to animal growth and development by modulating changes in metabolite enrichment and altering parameters of gut fermentation. This finding offers valuable insights into the management of lamb health and the development of targeted regulation strategies to enhance lamb productivity.

## Figures and Tables

**Table 1:** Comparison of dressing percentage and organ indices in HADG and LADG lambs.

Items	LADG	HADG	*p*-Value
Body weight, kg	25.80 ± 0.46 ^b^	40.91 ± 0.78 ^a^	*p* < 0.001
Carcass weight, kg	11.19 ± 0.26 ^b^	18.52 ± 0.19 ^a^	*p* < 0.001
Dressing percentage, kg	43.39 ± 0.61	45.39 ± 1.05	0.121
Heart index, %	0.43 ± 0.01	0.41 ± 0.01	0.246
Liver index, %	1.68 ± 0.08	1.54 ± 0.02	0.126
Spleen index, %	0.14 ± 0.01	0.13 ± 0.02	0.691
Kidney index, %	0.28 ± 0.01 ^a^	0.24 ± 0.01 ^b^	0.001
Lung index, %	1.85 ± 0.09	1.79 ± 0.09	0.518

Note: different lowercase superscript letters indicate significant differences on the same line, *p* < 0.05.

**Table 2:** Comparison of jejunal VFAs, digestive enzymes, and morphology in lambs.

Items		LADG	HADG	*p*-Value
VFA molar content	acetic acid, mmol/L	22.84 ± 1.40 ^a^	13.66 ± 1.58 ^b^	0.001
	propanoic acid, mmol/L	4.87 ± 0.37 ^b^	7.13 ± 0.53 ^a^	0.004
	butyric acid, mmol/L	4.35 ± 0.43 ^b^	5.78 ± 0.40 ^a^	0.029
	TVFAs, mmol/L	33.65 ± 2.07	28.15 ± 1.55	0.052
VFA molar ratio	acetic acid/ propanoic acid	4.75 ± 0.18 ^a^	2.05 ± 0.34 ^b^	0.000
	acetic acid ratio, %	67.89 ± 0.53 ^a^	47.39 ± 3.52 ^b^	0.001
	propanoic acid ratio, %	14.44 ± 0.60 ^b^	25.80 ± 2.03 ^a^	0.001
	butyric acid ratio, %	12.89 ± 0.85 ^b^	20.95 ± 1.64 ^a^	0.001
Digestive enzymes	beta-glucosidase, ng/L	87.07 ± 9.62 ^b^	304.52 ± 38.52 ^a^	0.001
	MCC, pg/mL	21.00 ± 1.30 ^b^	33.95 ± 3.34 ^a^	0.006
	xylanase, pg/mL	52.62 ± 6.32 ^b^	91.17 ± 11.06 ^a^	0.011
	CMC, pg/mL	16.46 ± 2.08 ^b^	55.58 ± 6.47 ^a^	0.000
	lipase, ng/mL	55.27 ± 2.22	62.62 ± 4.20 ^a^	0.144
	AMS, umol/L	6.36 ± 1.29 ^b^	20.92 ± 2.37 ^a^	0.000
Morphology	villus height, mm	0.61 ± 0.06	0.63 ± 0.07	0.842
	crypt depth, mm	0.34 ± 0.02	0.42 ± 0.04	0.062
	villus height/crypt depth	1.81 ± 0.14	1.51 ± 0.10	0.097

Note: different lowercase superscript letters indicate significant differences on the same line, *p* < 0.05. Abbreviations: MCC: microcrystalline cellulase; CMC: carboxymethyl cellulase; AMS: amylase.

**Table 3:** Analysis of rumen microbiota alpha diversity in lambs.

Items	LADG	HADG	*p*-Value
ACE	783.3619	862.2128	0.508
Chao1	782.4988	861.5077	0.507
Simpson	0.953113	0.9654	0.634
Shannon	7.107163	7.371238	0.646

## Data Availability

Data will be made available upon contacting the author.

## Supplementary Materials

The following supporting information can be downloaded at: https://www.mdpi.com/article/10.3390/ijms252413386/s1.

## Abbreviations

AA	Acetic acid
AA:PA	Acetic acid/propionic acid
AAR	Acetic acid ratio
AMP	Adenosine monophosphate
AMP/ATP	Adenosine monophosphate/adenosinetriphosphate
AMPK	AMP-activated protein kinase
AMS	Amylase
ASVs	Amplicon Sequence Variants
BA	Butyric acid
BAR	Butyric acid ratio
BW	Body weight
CMC	Carboxymethyl cellulose
CW	Carcass weight
DP	Dressing percentage
GLU	Beta-glucosidase
KEGG	Kyoto Encyclopedia of Genes and Genomes
LEfSe	Linear discriminant analysis (LDA) effect size
MCC	Microcrystalline cellulose
OPLS-DA	Orthogonal projections to latent structures discriminate analysis
PA	Propionic acid
PAR	Propionic acid ratio
TVFAs	Total volatile fatty acids
VFAs	Volatile fatty acids
Xyl	Xylanase
