# Supplementary figures and images for: Interaction Between Liver Metabolism and Gut Short-Chain Fatty Acids via Liver–Gut Axis Affects Body Weight in Lambs
Source: Int J Mol Sci. 2024 Dec 13;25(24):13386. doi: 10.3390/ijms252413386 (PMC11676651; doi:10.3390/ijms252413386)

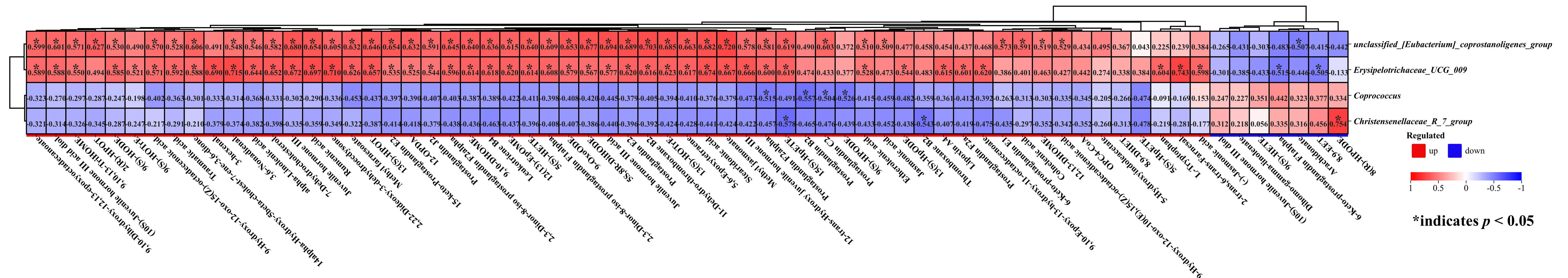

Supplement: Supplementary file 1 [file ijms-25-13386-s001.zip › Supplementary Figure S1Heat map analysis of liver KEGG top 5 networks enriched differential metabolites correlation with genus-level microbiota markers of HADG and LADG lambs..tif]

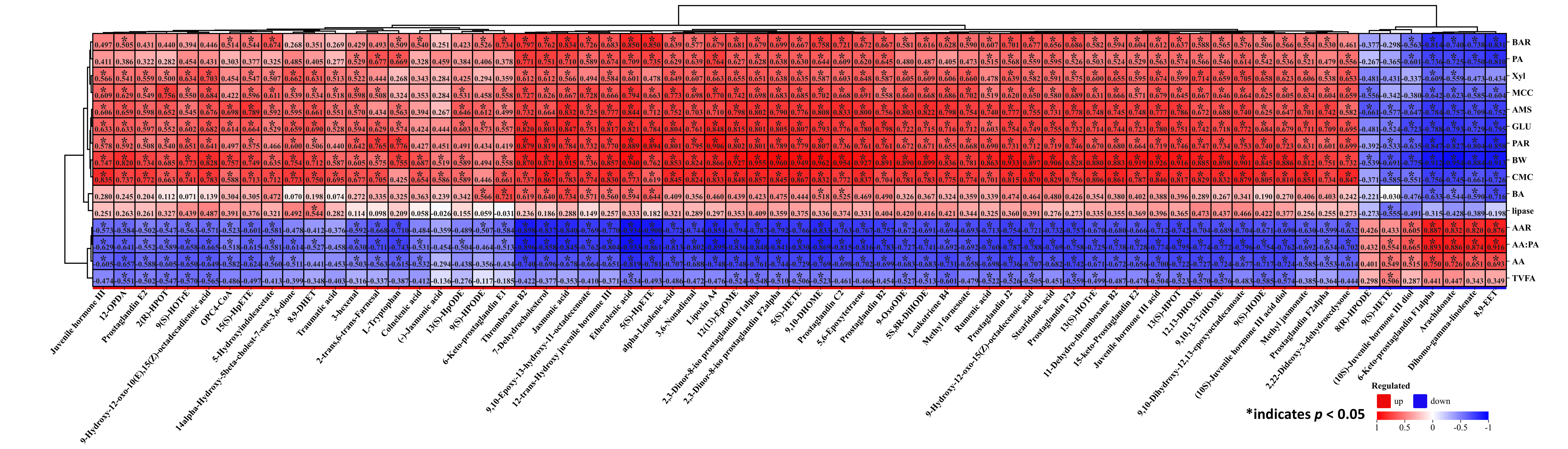

Supplement: Supplementary file 1 [file ijms-25-13386-s001.zip › Supplementary Figure S2Heat map analysis of liver KEGG top 5 networks enriched differential metabolites correlation with phenotypeof HADG and LADG lambs.tif]

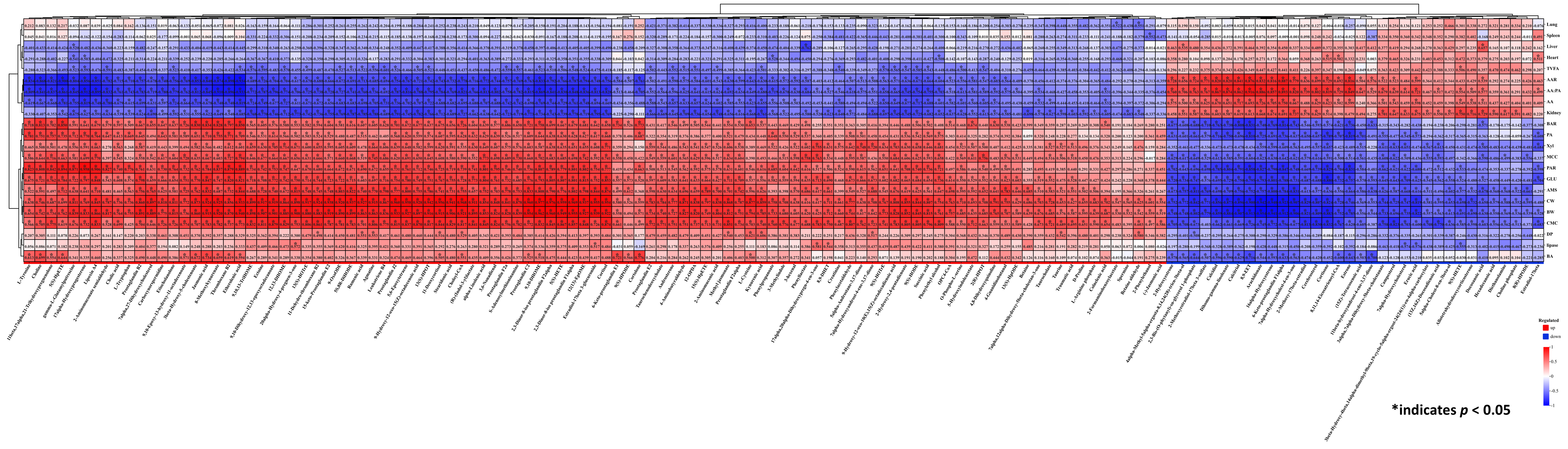

Supplement: Supplementary file 1 [file ijms-25-13386-s001.zip › Supplementary Figure S3 Heat maps were used to analyze the correlation between phenotype and major enrichment differences in KEGG regulation in liver.tif]

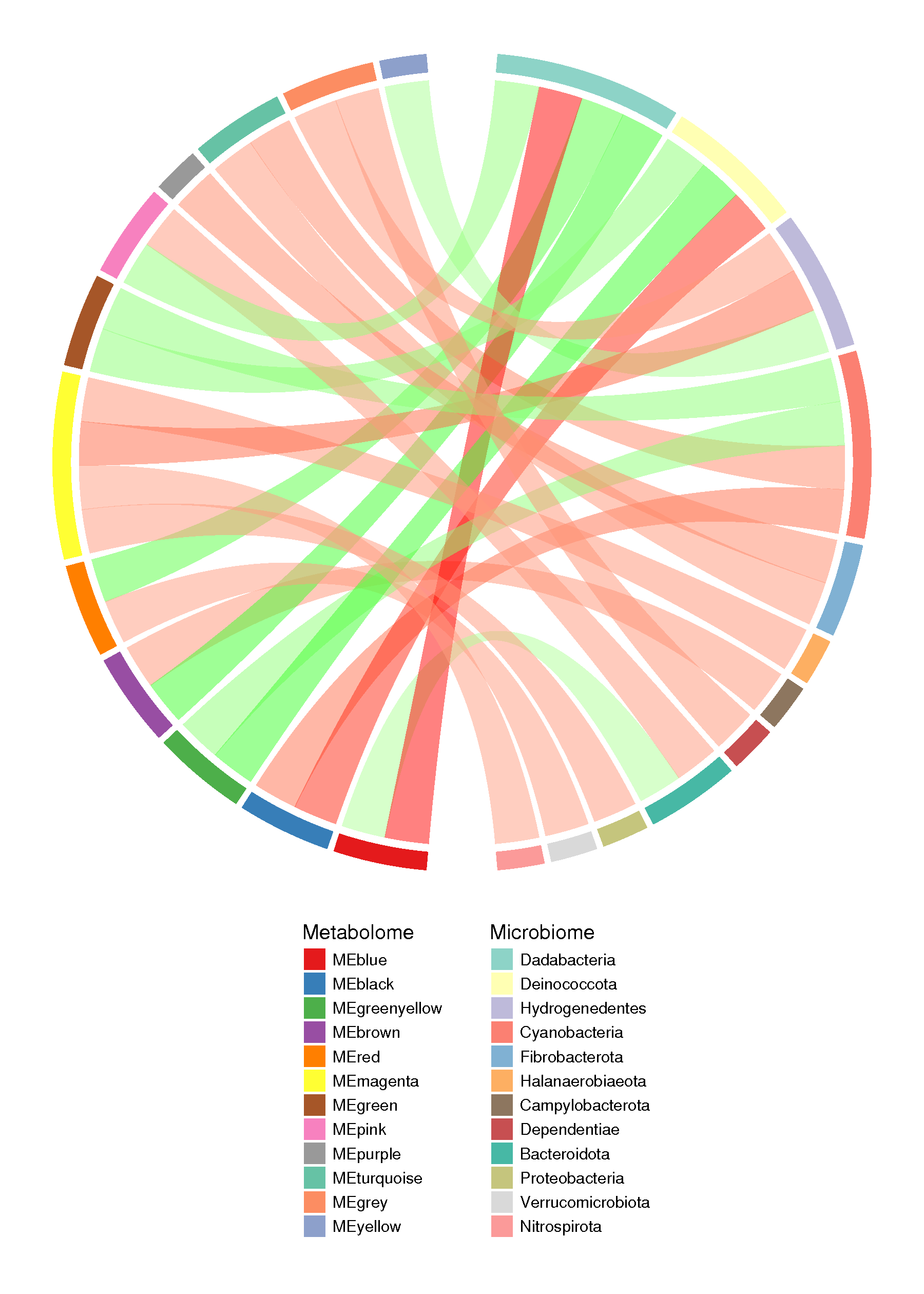

Supplement: Supplementary file 1 [file ijms-25-13386-s001.zip › Supplementary Figure S4 Metabolite module- jejunal phylum level microbiota chord diagrams.png]

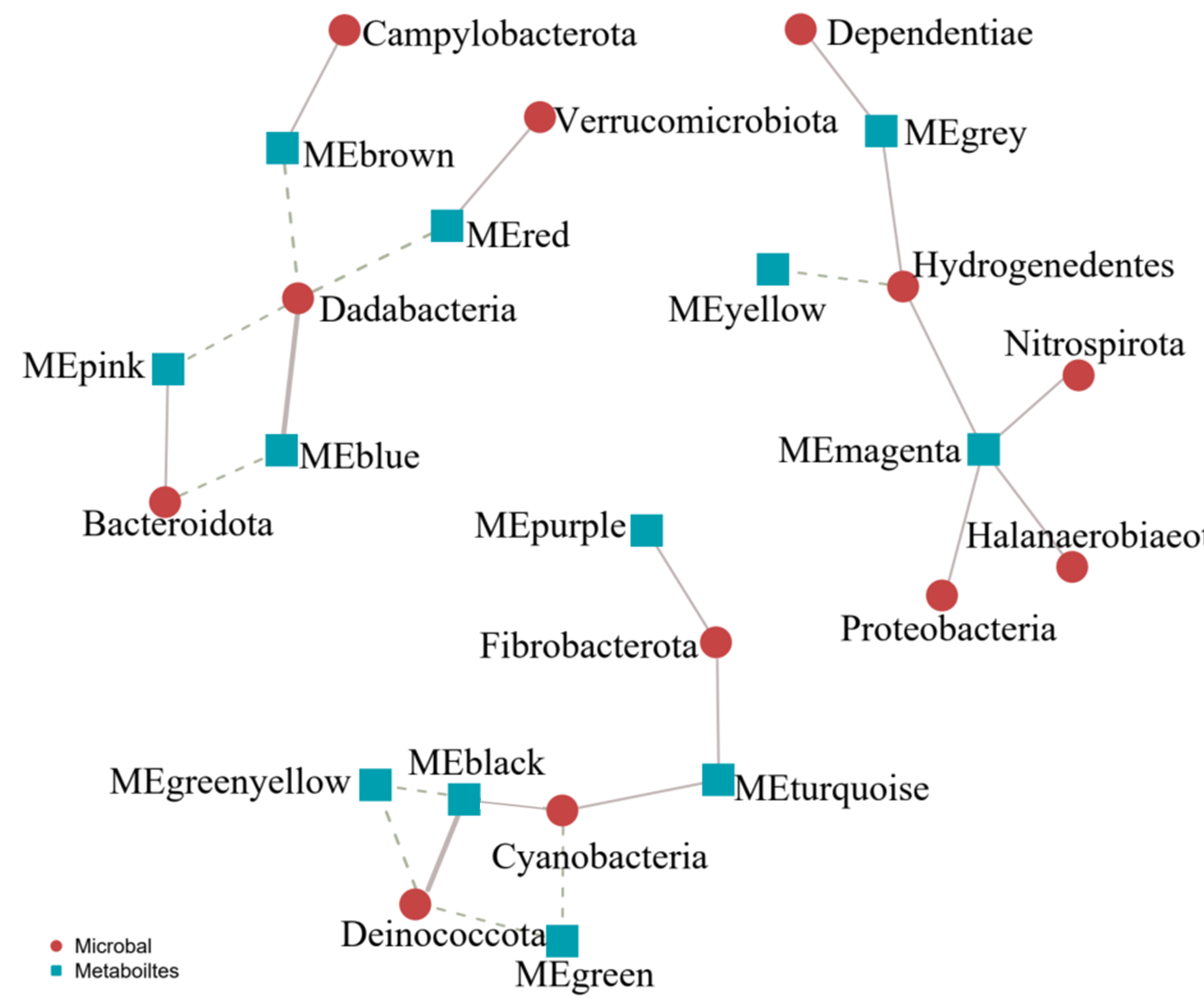

Supplement: Supplementary file 1 [file ijms-25-13386-s001.zip › Supplementary Figure S5 Metabolite module- jejunal phylum level microbiota correlation network diagrams..png]

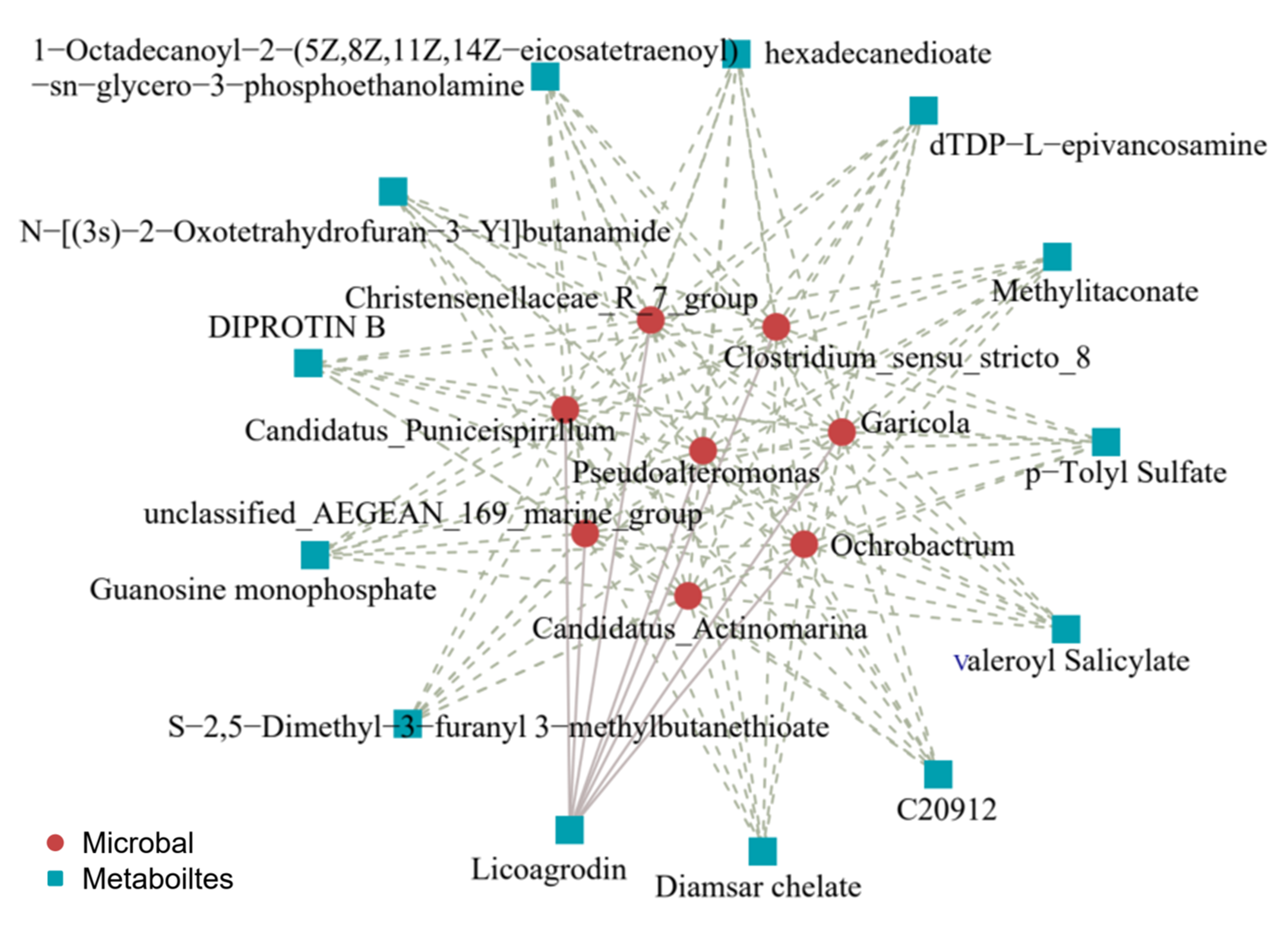

Supplement: Supplementary file 1 [file ijms-25-13386-s001.zip › Supplementary Figure S6 Liver difference metabolite and genus level difference microbiota correlation network diagrams.png]

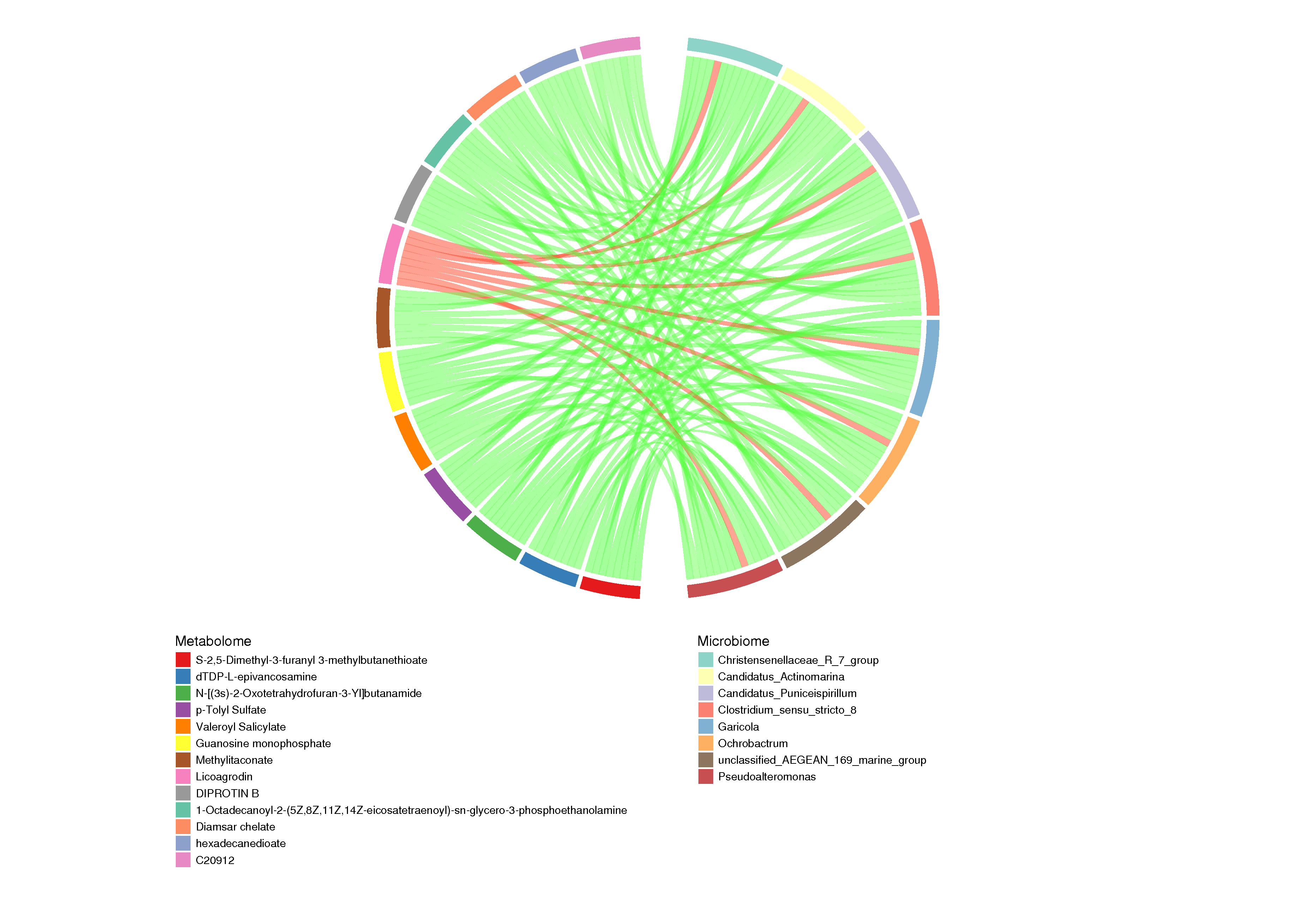

Supplement: Supplementary file 1 [file ijms-25-13386-s001.zip › Supplementary Figure S7 Liver difference metabolite and genus level difference microbiota chord diagrams.png]
